# Fruits, Spices and Honey Phenolic Compounds: A Comprehensive Review on Their Origin, Methods of Extraction and Beneficial Health Properties

**DOI:** 10.3390/antiox13111335

**Published:** 2024-10-31

**Authors:** Dimitrios G. Lazaridis, Apostolos-Panagiotis Kitsios, Antonios S. Koutoulis, Olga Malisova, Ioannis K. Karabagias

**Affiliations:** Department of Food Science and Technology, School of Agricultural Sciences, University of Patras, G. Seferi 2, 30100 Agrinio, Greece; up1076474@ac.upatras.gr (D.G.L.); up1076558@ac.upatras.gr (A.-P.K.); up1076552@ac.upatras.gr (A.S.K.); omalisova@upatras.gr (O.M.)

**Keywords:** phenolics, fruits, spices, honey, extraction, antioxidant activity, metabolism

## Abstract

Numerous health benefits have been attributed in the last decades to the regular consumption of fruits, vegetables, herbs and spices, along with honey, in a balanced diet. In this context, the aim of the present review was to provide the literature with the most relevant studies focusing on the determination protocols of these polyphenols and other reducing agents in selected fruits (orange, lemon, grapefruit, prunus, apricot, peach, plum, sweet cherry), spices (oregano, cinnamon, clove, saffron, turmeric) and honey of different botanical origin (nectar or honeydew). In addition, the content and the extraction methods of these compounds, along with their metabolic pathway, have been critically evaluated and discussed. Results showed that all fruits, spices and honey exhibit a considerably high antioxidant activity, which is mainly owed to their phytochemical content. Therefore, a balanced diet consisting of the combination of the foods studied herein may comprise a shield against chronic and other pathophysiological disorders and may be achieved through consecutive educational programs for consumers at an international level.

## 1. Introduction

In the last decades, research interest in polyphenols originating from plants has been increased. Fruits, spices and honey have an important role in the human diet around the world, but especially in Mediterranean countries, where phenolic compounds from plant products and honey are mainly associated with their culture. The terms “natural lifestyle” and “organic” are heard more often in our everyday lives, and this is happening in the effort of health organizations around the world to promote a healthier nutritional plan [1]. Young populations around the world have serious health problems originating from obesity, while psychological, metabolic, orthopedic and endocrine disorders are significantly higher in young people (adolescents and children) with excess weight compared to those of normal body weight [2]. Over the last three decades, fast food has entered people’s lives as a fast and tasty food source. The caloric intake of adolescents from fast food sources has increased seriously, resulting in obesity in teens and childhood and making it one of the most important problems worldwide in the current century [3]. Different studies have shown that more than 50% of college students and 30% of children eat fast food daily, while more than 33% of adults in the USA and 17% of children and teenagers are obese [4,5,6]. Eating fast, junk and processed food has consequences on human health, causing diabetes, obesity, heart attacks and cancer, while many people don’t realize or ignore these problems. Sweetened baked products, sweets, candies, and processed food are linked with brain cell destruction and intelligence lowering, leading to serious illness. World Health Organization (WHO) suggests a healthy diet, including vegetables, nuts, beans, seeds and fruits and decreased intakes of salt, excess sugar and saturated fat, as well as animal products that may lead to chronic disease and premature aging [7]. Processed foods contain excessive free oxygen radicals, and their consumption causes many pathological disorders, such as cataracts, cardiovascular diseases and aging. It is proven that the consumption of natural antioxidants from fruits, vegetables and spices can prevent these diseases. The main antioxidant compounds are polyphenols, and their feature is to enter into redox reactions, giving electrons (H^+^) from their hydroxyl groups occur in the para- or ortho-position [8]. Fruit antioxidants have defensive mechanisms that act against illness, particularly vitamin C and flavonoids that provide good health and mental stability. Oranges, grapefruits, berries, apples, grapes and aronia are some of the healthier fruits, rich in polyphenols and flavonoids, commonly suggested by WHO to be part of a balanced and healthy diet [8,9]. Among the numerous polyphenols found in fruits, the most common are hydroxycinnamic acids (i.e., sinapic, caffeic, ferulic, chlorogenic, para-coumaric) [10,11], flavanones (i.e., hesperetin, naringenin, naringin, narirutin, neohesperidin, poncirin, didymin, eriodictyol, isosakuranetin) [10,11,12,13,14], flavonols (i.e., quercetin, kaempferol, rutin) [15,16], flavonols (i.e., epicatechin, catechin, proanthocyanidins) [17,18], anthocyanins (i.e., cyanidin 3-rutunoside, cyanidin 3-glucoside, peonidin, pelargonidin 3-O-rutinoside) [19,20]. A critical parameter to discuss is the role of specific fruit parts in the polyphenol content. Indeed, different fruit parts such as peel, pulp or juice have different polyphenol content [21,22,23,24,25].

In parallel, spices are some of the richest foods in polyphenols and have an important role in cuisines around the world. Their essential oils contain high concentrations of aromatic hydrocarbons, esters and aldehydes, as well as secondary metabolites such as polyphenols and terpenoids [26]. Cinnamon, oregano, turmeric, saffron, and clove are some of the most common spices used, containing large quantities of phenolic compounds with strong antioxidant activity, such as cinnamaldehyde, curcumin, eugenol, gallic acid, quercetin and caffeic acid or its derivatives, along with hydroxybenzoic acids (vanillic acid, syringic acid, para-hydroxybenzoic acid, protocatechuic acid) [27,28,29]. Lastly, honey is a natural food produced by honeybees, converting honeydew or flower nectar into honey through the enzymatic conversion of sugars [30]. Honey consists of water, carbohydrates, amino acids and various phytochemicals, among them syringic, caffeic, and para-coumaric acid, but it also is rich in flavonoids such as quercetin, kaempferol and pinocembrin [30,31]. What is important to stress is that the honey botanical origin greatly affects its polyphenol content and beneficial health properties [32,33]. From an analytical point of view speaking, it is also basic to mention that the analytical instrumentation and the extraction methods used are crucial for the polyphenol content of foods, including fruits, spices, and honey. At the same time, an important feature holds the bioavailability of polyphenols in the human body. 

**Figure 1:**
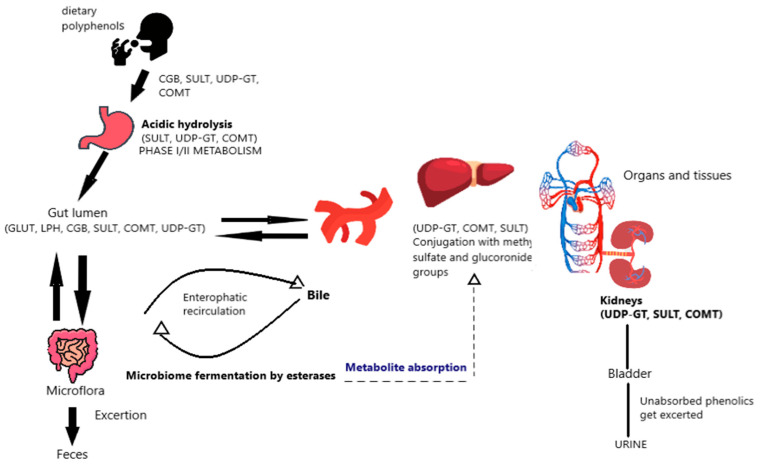
Bioavailability mechanism of dietary polyphenols.

Regarding the bioavailability of dietary polyphenols, when phenolic aglycones are compared to the same flavonoids that are glycosylated and found in various food matrices, they can be better obtained by intestinal barriers, improving their bioavailability [34]. The second phase of metabolism, which the flavonoids enter after being absorbed by the intestinal epithelium but before entering the bloodstream, results in the formation of various conjugated products. Specifically, sulfotransferases (SULTs) produce sulfates, uridine-5′-diphosphate glucuronosyltransferases (UGTs) enable the formation of glucoronides, and catechol-O-methyltransferases (COMTs) produce methylated derivatives. Furthermore, certain proteins linked to multi-resistance (MRP1, MRP2), which are involved in the third stage of flavonoid metabolism, mediate this metabolic biotransformation that impacts the distribution, absorption, and bioavailability of flavonoids at the cellular and tissue levels [35]. MRP2 carries flavonoids back into the intestinal lumen and is found in the apical membrane of the small intestine’s epithelial cells. MRP1 facilitates the movement of flavonoids within blood cells and is found in the vascular pole of enterocytes [36]. Additionally, these substances are transported through the portal venous system with the assistance of MRP3 and the glucose transporter (GLUT2); once inside, the metabolites swiftly arrive at the hepatocytes, where the aglycones are transferred to the peroxisomes and the Golgi apparatus, where they undergo additional metabolic processes [37]. Certain flavonoids include sugars that are resistant to the effects of LPH and CBG. As a result, they bypass the small intestine and enter the colon, where enterobacteria in the area can deglycosylate them. Aglycones are also converted by the colon’s microbiome into a variety of compounds that either get removed from the body by feces or taken up by the liver during enteropathic recirculation of bile excretion, where they are then further conjugated by certain enzymes, as previously mentioned [38]. However, several organic acid transporters secrete other metabolites into the systemic circulation following the metabolic changes that occur in hepatocytes. These metabolites are then either absorbed by cells or tissues or eliminated by the kidneys [30,39]. Figure 1 represents the discussed bioavailability mechanism of dietary polyphenols.

Considering the above, the present review article focuses mainly on phenolic compounds contained in different fruits, spices and honey, analyzing their action mechanism as natural antioxidants. It also summarizes the extraction methods used for the determination of phenolic antioxidants and several in vitro results from multiple studies in a comparative way and characterizes the importance of polyphenols in human health, preventing people from different chronic diseases.

## 2. Fruits

### 2.1. Citrus

Plant polyphenols are valuable components that are present in different types of fruits. The consumption of these phytonutrients has various positive benefits for human health; many of them include cardioprotective and anticarcinogenic actions. The presence of these compounds in citrus fruits is directly related to their antioxidant activity. Table 1 shows the total phenolic and total flavonoid contents of 3 citrus cultivars, along with the content of these compounds in different fruits part, and the extraction methods used for the determinations.

### 2.2. Orange

Orange (*Citrus sinensis* (L.) Osbeck) is a great source of polyphenols, rendering it a food of high nutritional value. Polyphenols that are present in oranges are responsible for different attributes. For example, anthocyanins are responsible for the formation of red color in different parts of blood orange, such as the pulp and the peels of the fruit [41]. Furthermore, the high antioxidant activity of orange juice promotes the protection of health [42]. Many studies have been conducted in the last 30 years by scientists to quantify the antioxidant activity in foods. One of them showed that different commercial orange juices manifested a significant antioxidant activity, which was observed and calculated in vitro experimental systems [43]. The concentration of phenolic compounds in citrus peels is very high, according to different studies [44,45]. Additionally, byproducts from citrus processing, due to the large amounts of natural flavonoids these contain, make them an excellent source of polyphenols for multi-usage [46]. One of the main polyphenols present in orange juice are flavonoids and hydroxycinnamic acids (HCA). Among the flavonoid subgroups in oranges, the quantity of flavanones outperforms the rest of the subgroups, rendering them the major flavonoids in oranges [10]. Flavanones most commonly appear in the form of glycosides. Some of the most common flavanone aglycones are hesperetin, isosakuranetin, naringenin, and eriodictyol. However, their quantities in comparison with the quantities of glycosides are to a lesser extent [10]. The two major flavanone glycosides that appear in sour oranges (*C. aurantium*) are naringin and neohesperidin, whereas the main flavanone glycosides in sweet oranges (*C. sinensis*) are narirutin and hesperidin [12]. Flavanone glycosides of sour and sweet oranges play a significant role in sugar quantities, consequently affecting the taste of oranges and ergo, as well as the differences in taste between them [12]. Hesperedin, being one of the most abundant flavonoids in citrus, has a wide spectrum of uses because of its nutraceutical effects, e.g., analgesic, antihypertensive, and anti-inflammatory actions [10]. On the other hand, hydroxycinnamic acids appear in the form of esters of sinapic, ferulic, caffeic and para-coumaric acids [10]. According to previous studies [41], factors such as harvest date and the variety of orange played a significant role in the formation of total polyphenols in a wide plethora of orange juice samples (14 juice samples, N = 42). The results showed remarkable differences amongst the phenolic content of those samples; for instance, the total phenols varied from 361.4 ± 16.9 to 1147.2 ± 34.9 (µg/mL) ferulic acid equivalents. The total anthocyanins varied from 1.2 ± 0.19 to 278.4 ± 15.47 (μg/mL) as cyanidin-3-glucoside. The total flavanones content of orange juices showed notable differences as well. The highest values recorded were 444.5 ± 51.7 (μg/mL), with the most abundant flavanones being the hesperidin and narirutin, and the lowest values recorded were 150.2 ± 14.5 (μg/mL). Finally, the total hydroxycinnamic acids content values varied from 33.4 ± 4.9 to 140.2 ± 15.3 (μg/mL), represented by caffeic, ferulic, para-coumaric and synapic acids. Results of the study further supported the idea that apart from polyphenols, anthocyanins constituted an important factor that influenced the antioxidant activity of fresh orange juices. Whole fruits are recommended for consumption by dietary guidelines [47]. As such, citrus pulp can be a valuable source of phytonutrients for the human diet. Many studies have been carried out to quantify the phenolic content of citrus pulps and to compare the phenolic profiles of different parts of citrus [40,48]. The researchers used different extraction techniques, in combination with the usage of different organic solvents, to quantify the phenolic content of orange pulp and to estimate the antioxidant activity of orange pulp.

### 2.3. Lemon

Lemon (*Citrus limon* (L.) Burm. f.) is one of the main citrus species produced globally, after oranges and mandarins, according to the FAO (2020 statistical bulletin). Lemons are used in various sectors for their characteristic taste and unique properties, such as food preservatives, cook materials, beverages, etc. [49]. Lemons are also an important source of phytochemicals (phenolic compounds), vitamins, minerals, carotenoids, dietary fibers and essential oils. Their antioxidant activity is related to the existence of multiple chemical substances (vitamin C, phenolic compounds, etc.) that have antioxidant properties [50]. As a result, these components are related to beneficial health properties by preventing different types of diseases, such as cardiovascular diseases and diabetes [50]. According to Abad-García et al. [13], the most abundant flavanone in lemon juice was hesperetin-7-O-rutinoside, with eriodictyol-7-O-rutinoside being the next ample flavanone in lemon juice. The high content of eriodictyol-7-O-rutinoside is an index of a compound manifested mainly in lemon juices [13]. The most common flavanones in lemon peels are hesperidin and narirutin. The most ample flavonoid in lemon peels is eriocitrin, which manifests anti-inflammatory action in vitro and in vivo as well [51]. Many studies have proven the nutritional and health-promoting effects of lemons. Xi et al. [52] investigated the antioxidant capacity and phenolic profile of fruit parts from different lemon cultivars. The results of those studies showed multiple variations amongst the different cultivars and different parts of the fruit. Significant divergences of the total phenolic and total flavonoid content were reported, and the juice values ranged from 0.29 to 0.52 mg/g GAE FW and 0.26 to 0.44 mg/g RE, respectively. Peel total phenolic values varied from 3.17 to 4.63 mg/g FW, while total flavonoid values ranged from 5.12 to 8.30 mg/g FW. 

Pulp and whole fruit values on total phenolic content ranged from 2.43 to 3.46 mg/g FW and 1.63 mg/g to 3.04 mg/g FW. The total flavonoid content of pulp and whole fruit showed as well major differences, ranging from 3.86 to 5.38 mg/g FW and 3.16 to 9.27 mg/g FW, respectively. Based on the results of the aforementioned research, it can be reasonably deduced that peels contain the highest phenolic content. Similar experiments on lemon have been carried out to verify the concentration of total phenolic and flavonoid content in different parts of lemon, some of them reported in Table 1.

### 2.4. Grapefruit

The antioxidant capacity of grapefruits (*Citrus paradisi* Macf.) is related to their availability of natural antioxidant compounds, such as carotenoids, vitamins, phenolic compounds and limonoids [53]. These phytochemicals protect the organism from the oxidative damage caused by free radicals. Therefore, many diseases related to oxidative stress, such as cancer, cardiovascular diseases and diabetes, can be counteracted with the consumption of foods that have a high content of phytochemicals, reducing the chances of manifestation of the abovementioned diseases [54]. Two of the dominant groups of phenolic compounds in citrus fruit juices are flavanones and phenolic acids [55]. The most salient phenolic acid present in grapefruit juices is para-hydroxycinnamic acid, along with caffeic, ferulic, sinapic, chlorogenic and para-coumaric acids [11]. Another main subgroup of polyphenols that are present in grapefruits are flavanones, with the most major ones being narirutin and naringin [14]. Much research has been carried out to identify the flavanone glycosides present in grapefruit juice. The results showed that neohesperidin, poncirin, didymin, and hesperidin are the major components [56]. Moreover, flavanones manifest a wide range of therapeutical properties, including antihypertensive, anti-inflammatory, hypolipidemic, and diuretic properties [57]. Vincenzo et al. [58] investigated grapefruit juice from star ruby and marsh varieties to quantify different phenolic compounds in juice. The total phenolic content of marsh juice was 153.08 ± 2.01 mg L^−1^, whereas the star ruby juice recorded higher values at 167.22 ± 0.98 mg L^−1^. Total flavonoid content was higher in marsh juice at 390.21 ± 9.32 mg L^−1^, and in star ruby, it was 310.14 ± 4.12 mg L^−1^. At last, total anthocyanins content was higher in star ruby juice, and the recorded values were 1.87 ± 0.04 mg L^−1^, whereas in marsh juice, they were 0.42 ± 0.01 mg L^−1^. In other studies, the phenolic content of whole fruit, peel, and pulp of different grapefruit varieties was quantified, and the results are reported in Table 1.

### 2.5. Prunus

*Prunus* L. is a genus of plants of the Rosaceae family, which encompasses the stone fruits. The global cultivation of these fruits plays a major role in the domestic economies because of their wide plethora of uses. Approximately 200 species are included in the genus *Prunus*, with the most important stone fruits being plums, cherries, almonds, peaches, and apricots [59]. The categorization of the genus Prunus encompasses five subgenera: *Prunus* (*=Prunophora*), *Amygdalus*, *Padus*, *Leucocerasus* and *Cerasus* [60]. These fruits are rich in vitamins, polyphenols, pectin, and minerals, rendering them an exceptional source of phytochemicals. A noteworthy percentage of products are produced using them as the major ingredients, for instance, jams, dried fruit products or purees. The numerous phytochemicals of stone fruits provide protection against a wide range of diseases, including cardiovascular diseases, diabetes, and obesity [61]. 

#### 2.5.1. Apricot

Apricots (*Prunus armeniaca* L.), which belong to the subgenus prunus, are fruits with high nutritional value due to the existence of numerous phytochemicals [62]. Specifically, the pericarps of apricots are a natural source of organic acids, saccharides, mineral elements (potassium, iron, and boron), polyphenols and vitamins (Vitamins C, B, provitamin A) [61]. One of the major distinctive characteristics of apricots is their high phenolic content, which deems apricots as a natural functional food [63]. Factors such as the maturity of the fruit [15], apricot variety [64], cultivation systems and geographical origin [65] have a direct impact on the quantity of phenolic content. Research studies showed that there is a broad variety among the subgroups of the polyphenols present in apricots. Extensively, flavonoids such as flavonols, flavanols, and phenolic acids have been determined in apricots. The main flavonols occurring in apricots are rutinosides and glucosides of quercetin and kaempferol, and the most dominant one is rutin (quercetin 3-rutinoside) [15]. Continuing with flavonols, epicatechin and catechin are the major flavonols occurring in apricots, and it is extensively mentioned in the literature that the quantity predominance of one against the other depends on the variety of apricot [66]. Finally, the predominant phenolic acid in apricots is chlorogenic acid [15], with para-coumaric acid, gallic acid, caffeic acid, and neochlorogenic acid being present in lesser amounts, however, affected by the apricot variety [66]. It is worth mentioning that polyphenols in apricots are mainly responsible for the antioxidant activity of the fruit and play a major role in the formation of color and taste of fruit [67]. The nutraceutical properties of apricots are associated with the actions of bioactive substances such as polyphenols. Many health-providing effects are manifested by the consumption of apricots, including anti-hypertensive, anti-inflammatory and hepatoprotective effects [67]. Table 2 shows the total phenolic, total flavonoid and specified phenolic compound concentrations in different apricot varieties and fruits parts.

#### 2.5.2. Peach

China is the native region of peach (*Prunus persica* L.), one of the most popular stone fruits, which belongs to the subgenus *Amygdalus*. Its ever-increasing popularity relies on different organoleptic and nutrition-based parameters [69]. Different groups of polyphenols have been determined in peaches, including phenolic acids, anthocyanins, flavonols, and flavonols [69]. It is noteworthy that the phenolic content in the pericarp is higher than that of peach flesh in general [19]. Amongst the polyphenol subgroups, the hydroxycinnamic acids are the most prevailing among others, with the representative acids being chlorogenic and neochlorogenic [70]. Based on previous studies, the abovementioned acids are the most opulent components in peaches [71]. Variations occur between different peach cultivars in flavonols, anthocyanin, and flavan-3-ol contents [17]. The prevalent flavonols identified in peaches are the glycosylated forms of kaempferol and quercetin derivatives [71], and there are also notable contents of rutin as well [69]. The most frequently appearing flavan-3-ols in peaches are catechin and epicatechin in both the peel and flesh of peaches [69]. Additionally, peaches contain a remarkable amount of proanthocyanidins (PACs) which appear in the form of polymers or oligomers of polyhydroxy flavan-3-ol units, e.g., (−)-epicatechin and (+)-catechin [18]. For example, ample amounts of proanthocyanidin B1 are mostly found in peach peels and flesh [69]. The phenolic compounds of peaches are correlated with their antioxidant activity, as proved by many studies in ex vivo and in vitro measurements [72,73]. Phenolic compounds are the representative antioxidant phytochemicals in peaches. Nonetheless, other phytochemicals such as carotenoids and vitamin C boost peaches’ antioxidant activity [57]. A considerable number of biological activities, namely, anti-inflammatory and anti-allergic activities [74], antioxidant activity [75], and anticancer and chemopreventive activities [76,77], are owed to phenolic compounds in peach. Some studies in the literature investigated the correlation between the phenolic content and the ripening of the fruit and how the first parameter is affected by the other. According to the experimental results of Li et al. [78], the phenolic content of peaches and its reduction is affected by the ripening stage. Specifically, the TPC values of peach extracts ranged from 75.5 to 46.4 mg GAE/100 g. Other researchers [71] measured the total phenolic content of different peach cultivars to verify the differences among them. The results showed that Spring Belle extracts had the highest amount of phenolic compounds (81.5 mg/g), with the Cardinal, Dixired, and Red Top being the following cultivars with the next higher concentrations (34.3 and 37.9 mg/g, respectively). Lastly, Flavorcrest and Romea had the lowest amount of phenolic compounds (23.1 and 19.8 mg/g, correspondingly).

#### 2.5.3. Plum

Plum (*Prunus domestica* L.) is a stone fruit of the Rosaceae family, which falls within the subgenus prunus. From a general perspective, plums are popular in the mass market for their nutritional benefits, unique taste, and the processing capabilities that they provide, which in turn contributes to the production of a broad selection of products originating from the raw material, for example, alcoholic drinks, juices, jams, fruit drinks and dried fruits [79,80,81]. Plums are particularly rich in polyphenols, as stated by many studies, and their concentration is largely affected by the cultivar [16,82]. As such, many scientists are engaged in researching plum polyphenols, and their findings specified that the main polyphenols in plums are flavonols glycosides, flavonols, hydroxycinnamic acids, procyanidins and anthocyanins [82,83]. The most common phenolic compounds in plums are the phenolic acids that hydroxycinnamic acids derive from, with neochlorogenic and chlorogenic acids representing the majority of phenolic acids [19]. In fresh plums, the hydroxycinnamates have been determined entirely in their esterified forms [16]. Subsequently, rutin (quercetin 3-rutinoside) is the most dominant in comparison to other flavonol glycosides in plums [16]. Except for the yellow plums, all plum cultivars encompass anthocyanins, such as cyanidin 3-rutinoside and cyanidin 3-glucoside [19]. The high polyphenol content of plums plays a crucial role in the potency of antioxidant activity [82]. Plum phytochemicals have vital nutraceutical properties that can effectively protect human health; many of them include the prevention of heart diseases and cancer, osteoporosis, and digestive-related illnesses [80]. Table 3 represents the TPC, TFC and total anthocyanins concentration of different plum varieties, along with their extraction methods.

#### 2.5.4. Sweet Cherry

The Rosaceae family encompasses an extensive selection of fruits. The sweet cherries (*Prunus avium* L.) appertain in the subgenus Cerasus, and the popularity of these foods is associated with their numerous advantages [85]. Sweet cherries are used to produce a variety of products such as alcoholic beverages, juices and jams, and their organoleptic characteristics (sourness, color, sweetness) are the leading factors that can entice consumers and boost their acceptance over the different products [85]. From a nutraceutical point of view, the consumption of sweet cherries is correlated with health benefits [86]. Prevention of degenerative and chronic diseases is considered to be a substantial property due to the consumption of sweet cherries since these are foods that contain a remarkable amount of nutrients and antioxidant compounds [20]. Apart from the aforementioned phytochemicals, the health-promoting effects are related to the phenolic content of cherries. For example, the manifestation of cancer risk and cardiovascular diseases can be decreased with polyphenol intake [87]. Moreover, the extracts of sweet cherries are distinguished for their free radical scavenging properties that can boost the prevention of cell oxidative-related damage and, as a result, provide antitumoral and anti-inflammatory properties [85]. Finally, the ingestion of sweet cherries is linked to protection from some other diseases, such as diabetes, forestalling Alzheimer’s disease, and the reduction of blood pressure [88,89]. It’s vital to mention that the phenolic composition and antioxidant activity of sweet cherries are affected to a great degree by climatic factors [90]. The major phenolic compounds exhibited in sweet cherries are anthocyanins, and their main role is the red color formation in the flesh and skin of the cherries. From the consumer’s point of view, the darker the color of the cherry, the more mature it is and the better quality it provides [85]. The dark color of sweet cherries has a direct connection with the anthocyanin content [91]. The ample amounts of 3-O-rutinose and 3-O-glucoside of cyanidin renders them the predominant anthocyanins in sweet cherries. However, there are small amounts of 3-O-rutinose of pelargonidin or peonidin as well [20]. There have also been identified hydroxybenzoic acid derivatives and hydroxycinnamic acids in sweet cherries, the most abundant of which are coumaroylquinic and caffeoylquinic acids [20]. Lastly, the presence of flavonols and flavan-3-ols are of great significance in sweet cherries, and the main phenolic compounds in this category are quercetin-3-O-rutinose and epicatechin [85]. Table 4 shows some important information about different sweet cherry varieties.

## 3. Spices

Over 500 polyphenols have been identified in different plant-based products, such as coffee, cocoa, wine, tea, vegetables, fruits, herbs and spices [27]. Spices are parts of different plants that are dried and used to improve taste, color, aroma and generally, the organoleptic properties of foods [94]. Since ancient times, spices have been used as food preservatives and flavor enhancers in medicine, given that these contain considerable amounts of polyphenols that offer antioxidant activity [95,96]. Spice’s oil phase consists of different chemical constituents, such as ketones, aldehydes, esters, aromatic hydrocarbons, polyphenols and terpenoids. Like every food source, spices are complex matrices, and their chemical composition is affected by bioprocesses that take place, but it can be determined due to the biochemical development, according to Bostan et al. [26,97,98]. Polyphenols are secondary metabolites that consist of biologically active compounds [99]. It is well known that these compounds have anti-inflammatory and antilipidemic effects antimicrobial properties, are natural therapeutic agents against cancer and their antioxidant activity is mainly attributed to hydroxyl groups in their structure [28,100,101]. Phenolic compounds with one or more hydroxyl groups are the most common secondary metabolites in plant-based products and can be found in free or bound form [27]. Free phenolics do not react with other molecules and are easily isolated, given that these are soluble in organic or polar aqueous solvents, respectively. On the other hand, bound phenolics are trapped via covalent bonds with other macromolecules, but their therapeutic, antioxidant and anti-inflammatory properties are greater than free phenolics [27,28,102]. Furthermore, the main classes of phenolic compounds in plant foods are phenolic acids, which are, in general, phenolic compounds that have one carboxylic acid group and can be found at high concentrations in fruits, vegetables, seeds and spices. Phenolic acids generally perform higher in vitro antioxidant activity than antioxidant vitamins [103] and can be separated into two groups: hydroxycinnamic and hydroxybenzoic acids [104]. Hydroxycinnamic acids are derivatives of cinnamic acid and can be found in plants as esters with quinic acid or glucose, and the most abundant hydroxycinnamic acid is chlorogenic acid (a combination of a form of caffeic and quinic acids). Ferulic, para-coumaric, sinapic and caffeic acids are the most common hydroxycinnamic acids. Compared to hydroxycinnamic acids, hydroxybenzoic acids are derived from benzoic acid. They have a common structure of C_6_-C_1_ and can be found conjugated with organic acids or sugars in soluble form, while the most common hydroxybenzoic acids are vanillic acid, syringic acid, para-hydroxybenzoic acid and protocatechuic acid [28,29].

There are thousands of different spices around the world that people use to improve the taste of their food, and some of them have health benefits due to the polyphenols that they contain [105]. Clove, oregano, cinnamon, rosemary, thyme, turmeric and saffron are some of the most popular and healthy spices that are a rich reservoir of phenolic compounds [106]. Among the different spices, unroasted green coffee beans contain a high concentration of total phenolics and caffeic acid (3,4-Dihydroxycinnamic acid) [98]. Table 5 shows the different solvents and extraction methods used for the determination of antioxidant activity, total phenolic content, total flavonoid content and caffeic acid contents on selected spices.

### 3.1. Oregano

Oregano (*Origanum vulgare*) is a woody plant with green leaves and is classified as the family of Lamiaceae. After harvesting, oregano gets dried, and the leaves crumble to use them in cooking. Oregano is known worldwide, especially in Mediterranean countries, for its aroma, bitter flavor and pungent taste [119]. The two main constituents of oregano and oregano oils are carvacrol (2-Methyl-5-(propan-2-yl) phenol) and thymol (5-Methyl-2-(propan-2-yl) phenol) are bioactive volatile compounds (monoterpenoid phenols) and well known for their antioxidant, anti-inflammatory, antitumor, antibacterial and antiparasitic properties [120]. Oregano also contains naringenin ((2S)-4′,5,7-Trihydroxyflavan-4-one) and pinocembrin (5,7-Dihydroxy-2-phenyl-2,3-dihydro-4H-chromen-4-one) [121], two flavanones from the flavonoid group of polyphenols [122]. Naringenin can also be found in citrus fruits [121], tomatoes [123] and specifically in Greek oregano [124]. In the study of Muchuweti et al. [107], oregano had the highest concentration of total phenolics among 9 different spices (oregano, cinnamon, sweet basil, bay leaves, mint, sage, rosemary, parsley, marjoram) and the highest antioxidant activity (61.76 ± 1.07%) to inhibit β-carotene solution. High-performance liquid chromatography (HPLC) analysis showed that oregano also contains para-hydroxybenzoic acid, para-hydroxybenzaldehyde, para-coumaric acid and ferulic acid. These results agree with the study of Assefa et al. [108], where methanolic extracts of 39 different spices were prepared, and the antioxidant activity of oregano leaves using the 2,2-diphenyl-1-picryl-hydrazyl (DPPH) assay was 53.90 mg of Trolox equivalents (TE)/g. Oregano was one of the top 5 most antioxidant spices in this study, between cloves, allspice, cinnamon, and marjoram. In 2007, Wojdyło et al. [109] resulted that oregano also contains high amounts of neochlorogenic acid and caffeic acid (96.3 ± 0.21 mg/100 g dw and 649 ± 0.07 mg/100 g dw, respectively), and high antioxidant activity, using 3 different methods, ABTS (2,2′-azino-bis(3-ethylbenzothiazoline-6-sulfonic acid) with 19.9 ± 1.00 μM trolox/100 g dw, DPPH (2,2-diphenyl-1-picryl-hydrazyl-hydrate) with 79.6 ± 2.04 μM trolox/100 g dw, and FRAP (Ferric reducing ability of plasma) with 405 ± 2.22 μM trolox/100 g dw. 

Ferulic acid (4-hydroxy-3-methoxy cinnamic acid) is an abundant phenolic phytochemical compound and can be found in seeds, oregano, barks and cereal leaves; it is a derivative of cinnamic acid and is classified as a hydroxycinnamic acid that interacts with polyamine lipids, oligosaccharides and polysaccharides [125]. Ferulic acid plays an important role in the rigidity of the cell walls of plants, which helps in the formation of other compounds like vanillic acid, synapic acid and curcumin [126]. It is observed that the highest antioxidant activity of cinnamic acid derivatives can be found in those that have a para-hydroxyl group. Furthermore, the key to stabilizing free radicals can be understood by the delocalization of π-type unpaired electrons on the aromatic rings, O atoms and double bonds, while the substitution of ortho-dihydroxy in the benzene ring seems to positively affect the neutralization ability of free radicals, making caffeic acid an antioxidant of hydroxycinnamic acids group [125]. 

### 3.2. Cinnamon

Cinnamon is received from the bark of *Cinnamomum* species and is one of the most known spices used as food and herbal medicine around the world. The cinnamon tree belongs to the Lauraceae family and has over 250 known species of the genus *Cinnamomum*, including *Cinnamomum cassia*, *Cinnamomum verum* (Ceylon) and *Cinnamomum burmannii* [98,127]. The main chemical compounds of cinnamon bark are cinnamaldehyde and its trans isomer, trans-cinnamaldehyde, along with eugenol in low concentration, which are contained in cinnamon’s essential oil and give its characteristic pungent flavor and odor. Also, cinnamon is a source of different flavonoids, like catechins and procyanidins, that belong to the group of flavan-3-ols. Finally different studies showed that cinnamon bark contains abundant phenolic compounds like catechin, epicatechin, protocatechuic acid, quercetin, para-hydroxybenzoic acid, syringic acid, para-coumaric acid, rosmarinic acid, caffeic acid and ferulic acid, that are responsible for the antioxidant, anti-inflammatory, anti-diabetic and cardiovascular effects of cinnamon [98,127,128]. It should be mentioned that cinnamon has a special mechanism with the ability to inhibit the release of fatty acids, such as arachidonic, which has an inflammatory effect and is associated with cardiovascular health [129]. The compounds contained in cinnamon bark can neutralize free radicals, chelate metal ions and enhance the activity of endogenous antioxidant enzymes. In the study of Roussel et al. [110], the total phenolic content of cinnamon extract was reported to be 82.3 mg of gallic acid equivalents (GAE)/g, with an EC_50_ value of 0.015 mg/mL, using the DPPH assay. This result is particularly impressive compared to other natural antioxidant spices, such as clove extract (*Syzygium aromaticum*), which contained 151.5 mg GAE/g with an EC_50_ value of 0.02 mg/mL, using the DPPH method again. While clove extract had higher phenolic content, the EC_50_ values indicated that cinnamon is more effective in free radical scavenging, demonstrating its strong antioxidant activity. However, the antioxidant activity depends on the extraction method and different parameters that are used (solvent, time of extraction, temperature etc.) [111]. Jayaprakasha et al. [112] reported that extraction with different solvents affects the total phenolic content of cinnamon extracts, and the best solvent to extract phytochemical compounds from cinnamon was water, followed by methanol, acetone and ethanol, respectively. Lazaridis et al. [98] developed a new and eco-friendly solvent produced by dry white wine distillation and extracted efficiently the phytochemical compounds from the equimolar mixture (10% *w*/*v*) of cinnamon/clove. The results of this study were unexpected and showed high antioxidant activity using the DPPH assay (55.12 ± 0.01%), low EC_50_ values in agreement with the studies mentioned above (0.08 ± 0.01%) and high total phenolic content (1120.24 ± 0.01 mg GAE/L), along with high quercetin content (566.77 ± 0.01 mg/L). In conclusion, cinnamon is a spice with high antioxidant activity and a high amount of phytochemical and phenolic compounds that can be extracted with different methods. 

### 3.3. Clove

**Figure 2:**
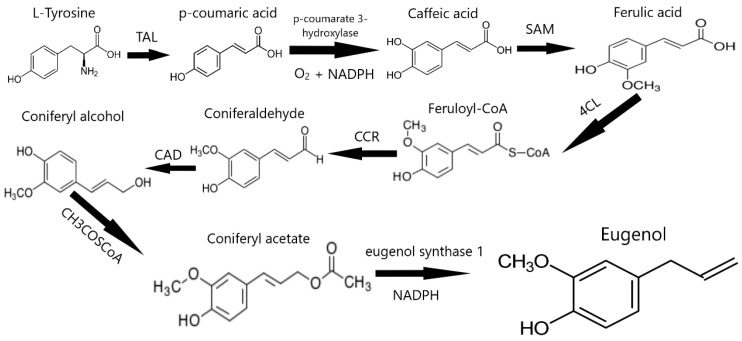
Synthesis of eugenol through biochemical reactions.

Clove (*Syzygium aromaticum*) is the aromatic flower bud of a tall tree in the family of Myrtaceae and can commonly be found in Asia, especially Indonesia, India, Pakistan and Africa. Clove is commonly used in many countries around the world for its special aroma, in meat flavors such as marinade, and in sweets. Clove contains bioactive compounds such as flavonoids, hydroxybenzoic and hydroxycinnamic acids, in combination with vitamins, carotenoids, phytosterols and polyphenols, which is attributed to its antioxidant, antimicrobial, anti-inflammatory and anticarcinogenic effects [89,130,131]. Moreover, the most dominant compound found in cloves is eugenol (4-allyl-2-methoxyphenol), a volatile phenolic compound in the class of phenylpropanoids, contained in clove bud oil and clove leaves oil at a concentration of 80–90% and has the strong and characteristic odor of clove [132]. Eugenol is produced through biochemical processes (Figure 2), starting with L-tyrosine converted to para-coumaric acid by the tyrosine ammonia lyase enzyme (TAL). Then, by para-coumarate 3-hydroxylase using O_2_ and NADPH, para-coumaric acid is converted to caffeic acid, methylated, and then to methylate caffeic acid by S-Adenosyl methionine (SAM), forming ferulic acid. Ferulic acid is converted to feruloyl-CoA using the enzyme 4-hydroxycinnamoyl-CoA ligase (4CL) and later reduced to conifer aldehyde by the reductase, cinnamoyl-CoA (CCR). Conifer aldehyde, in turn, is reduced to coniferyl alcohol by cinnamyl-alcohol dehydrogenase (CAD), which is transformed to coniferyl acetate by CH_3_COSCoA. Finally, eugenol is produced from the conversion of coniferyl acetate via the eugenol synthase 1 and NADPH [133,134]. 

The high antioxidant activity of clove and clove extracts is also attributed to eugenol due to its free hydroxyl group in its structure and can be found in a concentration range between 93.81 and 145.6 mg/g of fresh cloves [130]. As with coffee, the different harvest and after-harvest methods can effectively reduce the concentrations of eugenol in clove buds [98,135]. This was reported in the study of Lee et al. [136], where the eugenol concentration in dried clove buds was 24.371 mg/g. Another study showed that ethanol and water are better solvents for producing clove extracts than ethyl acetate, as they inhibited DPPH by 91.4% while ethyl acetate extract only 25.3%. Also, water and ethanol extracts had the highest total phenolic content (230 mg GAE g^−1^ extract and 293 mg GAE g^−1^ extract, respectively), unlike ethyl acetate extract, which resulted only in 58.5 mg GAE g^−1^ extract [114]. The antioxidant activity results of ethanol extract are totally in agreement with the study of Nassar et al. [114], where it was evidenced that ethanol extract of clove buds inhibited the DPPH by 93%. It is also remarkable that hexane extract resulted in 83% inhibition of DPPH, probably due to the high concentration of eugenol, eugenol acetate and thymol [114]. Furthermore, Widowati et al. [115] prepared ethanol clove extract at 13.29% *w*/*v* and reported that it had the highest antioxidant activity between 9 different extracts, near 95% and the lowest EC_50_ value of 0.004 mg/mL, along with the highest total phenolic content expressed in eugenol equivalent/mg (188.35). Finally, in comparison with the studies mentioned before, Rosarior et al. [116] proved that 20% *w*/*v* ethanol clove extracts inhibited the DPPH by 95%, and its EC_50_ value was 0.037 mg/mL. Clove ethanolic extract contained 250.93 ± 1.33 mg GAE/ g extract, in agreement with El-Maati et al. [113], while clove essential oil contained high kaempferol content (5.839 mg/g), as determined by HPLC coupled to diode array (DAD) analysis (HPLC-DAD). Kaempferol is a natural flavonol (3,4′,5,7-tetrahydroxyflavone) in the group of flavonoids and can be found in plants and plant-based products, especially in fruits and saffron, at high concentrations [116,137]. 

### 3.4. Saffron

Saffron is a spice collected from the dried stigmas of *Crocus Sativus* L. flowers, commonly known as saffron crocus, belonging to the family of Iridaceae. Saffron has been used since ancient times for its tonic and anti-fever properties, while recent studies indicate its anti-cancer and anti-inflammatory effects. The main producer countries are Greece, Spain, India, Morocco, France and Iran, which is the world’s leading producer with 90–93% of global production [117]. Saffron is the most expensive spice in the world, mainly used in high-end cuisines, especially for the unique yellow color that it gives. Saffron’s chemical composition is dominated by aldehydes and ketones, but also contains carotenoids (β-carotene), crocetin, picrocrocin ((4R)-4-(β-D-Glucopyranosyloxy)-2,6,6-trimethylcyclohex-1-ene-1-carbaldehyde) that is responsible for its pungent flavor), α-crocin (8,8-diapo-8,8-carotenoic acid) that gives its yellow-orange color and mainly contains safranal (2,6,6-Trimethylcyclohexa-1,3-diene-1-carbaldehyde), a cyclic terpenic aldehyde which is responsible for saffron aroma [138,139]. In their study, Kyriakoudi et al. [140] tried to quantify the percentage of crocin, picrocrocin and total crocins in aqueous methanol extracts in different ratios and how these are affected by the ultrasound-assisted method. Results showed that the best ratio to extract total crocins was 1:1 methanol and water for 30 min, providing a total amount of 39.4 ± 1.6% total crocins content in selected samples. On the other hand, the best ratio to extract total picrocrocin was 0.44% methanol for 30 min, providing a total amount of 19.3% total picrocrocin content in the same sample. These results demonstrate the high concentration of these phenolics in saffron samples. In 2017, Karabagias et al. [117] compared saffron samples from different countries (Greece, Spain, Iran, and Morocco) and different harvesting years to discriminate them geographically based on physicochemical and antioxidant activity parameters and volatile compounds analysis. Results showed that the highest antioxidant activity on DPPH assay and the highest total phenolic content was in the saffron samples from Iran, 39.49 ± 1.05% and 101.93 ± 11.11 mg GAE/g dry weight, respectively. Furthermore, it was observed that the results on Greek and Moroccan samples harvested earlier had significantly lower values on their antioxidant activity and total phenolic content, proving that storage can negatively affect the product’s antioxidant properties and total phenolic composition. Also, headspace solid-phase microextraction coupled to gas chromatography-mass spectrometry (HS-SPME–GC/MS) analysis showed that safranal was the major volatile constituent (79.04% in Moroccan samples), in total agreement with the previous and relevant study [139], but also reported high amounts of isophorone (3,5,5-Trimethylcyclohex-2-en-1-one), a cyclic ketone that has similar structure with safranal. Finally, carvacrol was identified in low amounts in Greek and Moroccan samples as a monoterpenoid phenol with anti-cancer and anti-inflammatory properties [120]. Mashoul et al. [141] in 2013, reported that the rich polyphenol and carotenoid content of saffron probably enable potential mechanisms that provide weight loss, and 4 years later, in 2017, this hypothesis was proved when Kotanidou et al. [142] demonstrated that saffron supplementation reduces the weight of adolescents with obesity. It should be noted that about 146,300 tons of saffron flower petals and stamens are discarded after the stigmas harvesting, leading to huge agricultural waste. In 2022, Lachguer et al. [118] prepared aqueous and organic extracts (diethyl ether, n-butanol, ethyl acetate, methanol) of flower waste and determined their physicochemical, antibacterial and antifungal properties. Results showed that diethyl ether extract had a significantly high concentration of total phenolics (214.29 ± 12.68 mg GAE/g dry matter) and a good concentration of total flavonoids expressed as quercetin equivalents (84.46 ± 16.10 mg of QE/g dry matter). Furthermore, in vitro antimicrobial and antifungal experiments showed that diethyl ether and ethyl acetate extracts exhibited microbial and fungal activities against *Staphylococcus aureus* and *Botrytis cinerea*, respectively. These results could lead to more studies to support the hypothesis that wasted saffron flowers might be natural antioxidants that can be used as food preservatives, supporting the zero-waste concept.

### 3.5. Turmeric

Turmeric is a spice produced by the root of *Curcuma longa*, a plant that belongs to the family of Zingiberaceae and is cultivated mainly in southern Asia due to its needs in rainwater and temperatures between 20 and 30 °C. After their harvesting, the roots boil for 30–45 min in water and then are dried in hot ovens. Finally, these are powdered, resulting in a yellow powder that people use for food coloring and taste. Later, this was assigned as a food coloring agent with the number E100 [143,144]. Turmeric’s major group of compounds are curcuminoids, which include curcumin, demethoxycurcumin and bis-demethoxycurcumin. Curcumin research is one of the favorite subjects of analytic, organic and inorganic chemists along with food scientists due to its documented pharmaceutical effects and its act as a therapeutic agent against chronic diseases, but also as an antioxidant that prevents lipid peroxidation, anti-angiogenic, anti-hyperglycemia and anti-proliferative effects, making curcumin one of the most studied chemical compounds [27,145]. Curcumin ((1E,6E)-1,7-bis(4-hydroxy-3-methoxyphenyl)-1,6-heptadiene-3,5-dione) is a ketone that contains two ferulic acid residues joined by a methylene bridge with two aromatic o-methoxy phenolic groups rings in its structure, connected by a seven-carbon linker and α, β-unsaturated β-diketone moiety, that interacts with protein thiols through Michael reaction, while β-diketone group reduces the metal-induced toxicity [146]. Also, curcumin can take enol form when the diketo-group exhibits keto-enol tautomerism. Turmeric can contain 2–9% curcumin depending on its harvesting and after-harvest processes, which can affect curcumin’s concentration, as mentioned before, for other spices [145]. Curcumin can be extracted from turmeric using different organic solvents such as ethanol, methanol, ethyl acetate, acetone and hexane, but also with Soxhlet extraction or ultrasonic extraction [145]. Curcumin’s action against lipid peroxidation is associated with the reduction of malondialdehyde (MDA) levels in the human body. Jakubczyk et al. [147] 2020 administered 308 people (60% women and 40% men) curcumin supplements of different concentrations on a daily basis and reported that MDA levels in their bodies decreased significantly by about 50%, supporting that hypothesis. As mentioned before, curcumin is one of the most studied phenolic compounds, so there are myriads of studies that prove its health benefits, not only in humans. Results from different studies in animals (rabbits, mice, rats) were gathered and proved that curcumin could reduce cholesterol and glucose levels but also reduce triglyceride levels [148]. Identification and characterization of curcumin in plants and plant extracts are carried out using spectroscopic methods like Fourier transform infrared spectroscopy (FTIR), nuclear magnetic resonance spectroscopy (NMR) and ultraviolet-visible spectroscopy (UV-VIS), where curcumin and curcuminoids present maximum absorbance at or around 425 nm on ethanolic extracts. Moreover, curcumin can be identified using chromatographic methods like gas chromatography or liquid chromatography coupled with mass spectrometry (GC-MS, LC-MS, HPLC), but the most common analytical technique for curcumin identification is liquid chromatography coupled with UV-VIS, providing the fingerprint absorbance at 420 nm [149]. Finally, among the curcumin derivatives, demethoxycurcumin and bis-demethoxycurcumin are in considerable proportions, as Jayaprakasha et al. [150] showed that commercial curcumin contains 77% curcumin, 17% demethoxycurcumin and 3% bis-demethoxycurcumin, while the antioxidant assay on ascorbic acid of these compounds, indicated that curcumin has higher antioxidant capacity, followed by demethoxycurcumin and bis-demethoxycurcumin, respectively. These results prove that CH_3_O in the curcumin structure plays a significant role in the antioxidant activity of curcumin.

## 4. Honey

In recent decades, there has been a frequent observation of degrading or chronic diseases such as cancer, hypertension, Alzheimer’s disease, diabetes, atherosclerosis and heart diseases. These health issues cause most deaths globally [151,152]. Recent studies suggest that there is a correlation between oxidative stress and the development and complications of the foretold disorders. The overpowering of the oxidants against the antioxidants is a way to describe the oxidative stress that leads to damage [153,154,155,156]. The effects of everyday life, such as physical damage, various infections, stresses, and toxic and carcinogenic compounds, to name a few, impact the human body as a result of biological and chemical degradation that later provokes peroxidation of cell membranes’ polyunsaturated fatty acids that liberates toxic substances such as free radicals. Research studies that examine the relationship between the mortality of the foretold health issues and the consumption of vegetables and fruits often find that the polyphenols that exist in their matrix drastically affect the morbidity from the diseases by reducing it [153].

Because of the data stated, the interest in researching natural bioactive substances increases more and more. That is also because, in many cultures, natural products such as bee products, fish oil, herbs, and more have been used as folk medicine for many centuries [154]. So, honey is a naturally formed product made from nectar or honeydew by honeybees that consist mainly of carbohydrates (monosaccharides, disaccharides, oligosaccharides and polysaccharides) [155], proteins and enzymes, amino acids, vitamins, organic acids, minerals, and Maillard reaction products and other phytochemicals [155,156]. Numerous research studies have reported that honey is a product that provides several benefits that affect human health, such as antibacterial [157], anti-fungal [158] and anti-inflammatory [159], reproductive [160,161], hypoglycemic, antioxidant [162], antihypertensive [163], gastroprotective [164], hepatoprotective [165] effects. It is a food product packed with phenolic acids and flavonoids that exert various biological effects with antioxidant activity being one of them. The season, the floral source, the geographic region, the environmental factors, the processing and the storage conditions are parameters that altogether contribute to these compounds’ composition and consequently to their antioxidant activity [154]. The above factors are responsible for both the botanical composition of the honey and its chemical composition, its flavor and other physical properties, and particularly its biological activity [166]. 

The total phenolic content of honey can reach up to 753 mg/g, which usually varies depending on the region. For instance, the famous Manuka honey from the blooms of New Zealand’s native bush has gained great fame because of its specific antibacterial properties that arise from the region’s climate and the chemical composition of the plant’s pollen. Some flavonoids and phenolic compounds that researchers have identified in honey are quercetin, chrysin, kaempferol, pinobanksin, pinocembrin, apigenin, luteolin, naringenin, gallic acid, *p*-coumaric acid, genistein, hesperetin, ferulic acid, syringic acid, ellagic acid, vanillic acid, and caffeic acid [32,167,168,169,170,171,172].

The aforementioned substances, as well as other phenolic compounds, have been designated as the only responsible compounds for the antioxidant and other medicinal effects of honey [50,172]. Antioxidants act by countervailing the oxidative deterioration caused by oxidants like O_2_, OH^−^, superoxide, and lipid peroxyl radicals. Generation of cytotoxic compounds, atherosclerosis, mutagen synthesis, diabetes, cancer and various other chronic and lingering diseases are directly affected by oxidative stress which cells oppose by creating a defense system. Catalase, peroxidase, superoxide dismutase, tocopherol, ascorbic acid and polyphenols are some of the antioxidant agents that make up the system [173] that provoke a chain reaction of stimulating molecules like proteins, nucleic acids, carbohydrates and lipids that later activate the antioxidant response which acts by forming less toxic or more stable molecules [32,174]. More specifically, phenolic substances neutralize free radicals by giving off hydrogen from one of the hydroxyl groups. So, the degree of antioxidant activity is dependent on the number of hydroxyl groups [175].

Adding honey (1.2 g/kg) to the everyday diet enhances both the amount and the activity of antioxidant substances such as vitamin C, beta-carotene, uric acid and glutathione reductase in healthy humans [33]. Ahmed and Othman, in a recent study [32], reported that Tualang honey (a Malaysian multifloral jungle honey) has antimicrobial, anti-inflammatory, antioxidant, antimutagenic, antitumor, and antidiabetic properties, along with wound-healing properties. In a more recent study [32], it was found that the results from testing various Greek types of honey (nectar and honeydew) regarding their total phenolic content (TPC) showed significant differences amongst each other, according to the statistical analysis. Samples from many Greek geographical regions (Epirus, Macedonia, Evros, Thessaly, Central Greece, Euboea, Peloponnese, Crete, Aegean Islands) and botanical origin (thyme, orange, heather, oak, pine, acacia, multifloral blossom) were collected and then subjected to analysis. It was also shown that there were no significant differences between Greek honey as a whole and other countries’ honey (samples from Bulgaria, Cyprus, the United States of America, and New Zealand).

In another study evaluating the characteristics and properties of Turkish nectar and honeydew honey, there was an indication that its color may have a correlation to its antioxidant activity and botanical origin [176]. Identifying the compounds that constitute honey can be a challenging analysis as it is a rich and complex matrix. Several analytical techniques and methods have been utilized to determine the diverse and complex phenolic profile of honey. The one that has been used routinely is high-performance liquid chromatography (HPLC) [177,178,179]. HPLC, in combination with diode array detection (DAD) profiling, has been chosen for several case studies about honey-type characterization and identification [173,176,177]. Some typical solvents for the extraction of polyphenols from honey are acidified water, water/methanol or ethyl acetate [32,176,180]. 

**Figure 3:**
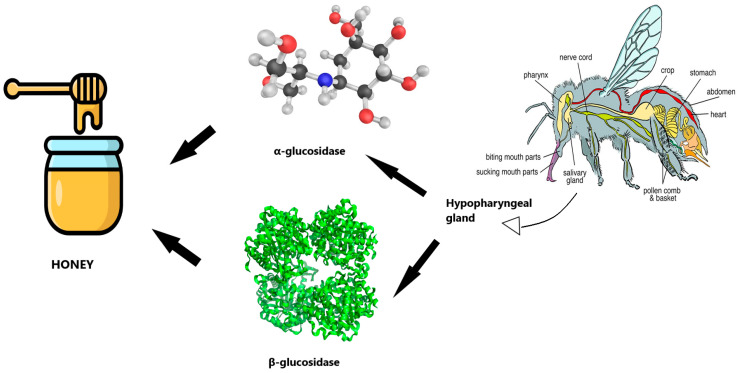
Contribution of honeybee organs to honey production and phenolic aglycones formation.

Studies on flavonoids dominate the field of phenol metabolism research. The hydrolysis process, which can be carried out by two different types of enzymes found in the small intestine of honeybees, as well as bacterial enzymes found in the intestine of the honeybees, is the first stage of flavonoid metabolism. Two β-endoglucosidases, namely lactase phlorizin hydrolase (LPH) and cytosolic β-glucosidase (CBG), are capable of catalyzing the β-hydrolysis of the sugar in glycosylated flavonoids [181]. Because LPH, which is present in the brush border of enterocytes, catalyzes the hydrolytic reaction, the liberated aglycone has a higher lipophilicity and can penetrate epithelial cells more readily. During the hydrolysis reaction catalyzed by CBG, polar glucosides are delivered into epithelial cells via a sodium-dependent glucose transporter 1 (SGLT1), where they undergo hydrolysis [182]. According to some research, certain flavonoids can prevent monosaccharide diffusion into intestinal epithelial cells that are dependent on sodium (Na). When analyzing honey and its flavonoids, it’s important to keep in mind that some of the glucosidases in this matrix originate from the oral glands of bees (Figure 3). This could be a further pathway for the hydrolysis of these compounds and provide an explanation for why so many flavonoids in honey are found in the form of aglycones. 

## 5. Discussion

The analysis of over 180 articles in the present review paper showed some important key findings regarding the origin, methods of extraction and beneficial health properties of polyphenols in selected fruits, spices and honey. The topic addressed herein was selected, as mentioned before in the Abstract section, on the basis of a balanced diet (i.e., Mediterranean diet) principles focusing on specific foods, as not any review article previously has combined knowledge on the topic studied for these foods. What is important to stress is that fruits possess different polyphenols that are dependent on the type of fruit, the fruit’s part (peel, pulp, juice) and the analytical methodologies followed for the extraction and determination of these compounds. Indeed, if someone takes a deep look at Table 1, Table 2, Table 3 and Table 4, he/she will notice that the total phenolic, total flavonoids and specific phenolic compounds content in orange, lemon, grapefruit apricot, plum, and sweet cherry vary significantly and are dependent on the above-mentioned parameters [16,21,22,23,24,25,40,66,68,72,84]. The same holds also for the pigments of fruits (anthocyanins) and condensed tannins [91,92]. The development of accurate analytical and biochemical techniques has led to extensive knowledge about the content of these compounds in fruits and their bioavailability, which makes the organization of diets after regular consumption of these fruits affordable. In parallel, the use of spices in foods as alternatives to salt makes a clear contribution to the welfare of humans’ health and sustainability, given that the spices analyzed, such as oregano, cinnamon, clove, saffron, and turmeric, have considerable bioactive components such as polyphenols and terpenoids that possess antioxidant activity. As in the case of fruits, the analytical extraction methods followed (either solvents or extraction techniques) greatly affect the content and the antioxidant activity of these polyphenols [98,107,108,109,110,111,112,113,114,115,116,117,118], expressed either as total phenolic or total flavonoid compounds or specific phenolic compounds (Table 5). Another critical point to mention is the double role of spices, which is unique in nature. More specifically, spices are categorized into foods and, at the same time, can be used as natural preservatives of other foods, boosting at the same time their sensorial characteristics. In this context, we should not forget that the phenolic content of honey and its beneficial health properties are also influenced by its botanical origin, honeybee’s metabolism, and geographical features (region, climatic conditions, processing practices, etc.). In addition, the content and the determination of these polyphenols are affected by the applied extraction methods (mostly acidified water, water/methanol, or ethyl acetate) and analytical instrumentation [33,176,180]. Notably, honeydew honey has a higher polyphenolic content compared to nectar honey [33], and therefore, research on the beneficial health/medicinal properties of honeydew honey has greatly expanded in the last few years [32,33,180].

## 6. Conclusions and Future Perspectives

Polyphenolic compounds found in fruits, spices or honey are numerous. The fruit’s part, the type of spices and the botanical origin of honey (among others) play a crucial role in their content. We should not forget the extraction methods and the analytical instruments used to determine these phytochemicals. With the improvement of the analytical instruments and laboratory methodologies, new phytochemicals may be discovered in the near future. However, the topic has to do mostly with consumers’ knowledge about the beneficial effects of these compounds on the human body. Therefore, educational programs related to the good pathophysiological status of consumers by regular consumption of these foods should be exhaustively generated at an international level to protect humans from chronic or incurable disorders and thus, make a step forward to reach an increase in life expectancy. To our knowledge, there is not any review article in the recent literature analyzing critically these specific foods, contributing thus to the main purpose of the study and, at the same time, flourishing the existing literature.

## Figures and Tables

**Table 1:** Total phenolic and total flavonoid contents of different citrus cultivars (orange, lemon and grapefruit) in different parts of fruits and extraction methods.

Cultivar	Fruit Part	Solvent	Extraction	Total Phenolic Content	Total Flavonoid Content	References
*Citrus sinensis* (L.) Osbeck	Peel	Acetone	Soxhlet	114 mg GAE/g	-	[40]
*Citrus sinensis* (L.) Osbeck	Peel	Methanol	Soxhlet	158 mg GAE/g	-	[40]
*Citrus sinensis* (L.) Osbeck	Peel	Water	Soxhlet	210 mg GAE/g	-	[40]
*Citrus sinensis* (L.) Osbeck	Peel	Hexane	Soxhlet	79 mg GAE/g	-	[40]
*Citrus sinensis* (L.) Osbeck	Peel	Water	Aqueous	215 mg GAE/g	-	[40]
*Citrus sinensis* (L.) Osbeck	Pulp	Acetone	Soxhlet	522 mg GAE/g	-	[40]
*Citrus sinensis* (L.) Osbeck	Pulp	Methanol	Soxhlet	465 mg GAE/g	-	[40]
*Citrus sinensis* (L.) Osbeck	Pulp	Water	Soxhlet	330 mg GAE/g	-	[40]
*Citrus sinensis* (L.) Osbeck	Pulp	Hexane	Soxhlet	201 mg GAE/g	-	[40]
*Citrus sinensis* (L.) Osbeck	Pulp	Water	Aqueous	326 mg GAE/g	-	[40]
*Citrus limon* (L.) Burm. f.	Peel	n-Hexane	Reflux	8.9 ± 0.08 mg GAE/g	27.2 ± 0.03 mg QE/g	[21]
*Citrus limon* (L.) Burm. f.	Peel	Ethyl acetate	Reflux	13.8 ± 0.07 mg GAE/g	24.9 ± 0.40 mg QE/g	[21]
*Citrus limon* (L.) Burm. f.	Peel	Ethanol	Reflux	15.2 ± 0.02 mg GAE/g	28.9 ± 0.04 mg QE/g	[21]
*Citrus limon* (L.) Burm. f.	Pulp	n-Hexane	Reflux	1.4 ± 0.02 mg GAE/g	30.2 ± 0.20 mg QE/g	[21]
*Citrus limon* (L.) Burm. f.	Pulp	Ethyl acetate	Reflux	9.0 ±0.14 mg GAE/g	8.5 ± 0.10 mg QE/g	[21]
*Citrus limon* (L.) Burm. f.	Pulp	Ethanol	Reflux	14.7 ± 0.17 mg GAE/g	10.4 ± 0.07 mg QE/g	[21]
*Citrus limon* (L.) Burm. f.	Juice	-	-	64.5 ± 1.4 mg GAE/L	24.0 ± 0.01 mg GAE/L	[22]
*Citrus limon* (L.) Lisbon	Juice	-	-	730.46 mg GAE/L	211.36 mg CE/L	[23]
*Citrus limon* (L.) Eureka	Juice	-	-	690.62 mg GAE/L	219.27 mg CE/L	[23]
*Citrus limon* (L.) Mayre	Juice	-	-	825.37 mg GAE/L	216.61 mg CE/L	[23]
*Citrus limon* (L.) Bush	Juice	-	-	998.29 mg GAE/L	220.34 mg CE/L	[23]
*Citrus paradisi* Red	Whole fruit	80% aqueous methanol	-	23.83 ± 34.64 mg GAE/g	-	[24]
*Citrus paradisi* White	Pulp	80% aqueous methanol	-	16.96 ± 7.86 mg GAE/g	-	[24]
*Citrus paradisi* Green	Peels	80% aqueous methanol	-	20.87 ± 13.81 mg GAE/g	-	[24]
*Citrus paradisi* Red	Juice	-	-	359 ± 0.029 mg GAE/L	-	[25]
*Citrus paradisi* Red	Peels	Ethanol	UAE	283 ± 0.018 mg GAE/L	-	[25]
*Citrus paradisi* White	Juice	-	-	747 ± 0.098 mg GAE/L	-	[25]
*Citrus paradisi* White	Peels	Ethanol	UAE	192 ± 0.015 mg GAE/L	-	[25]

GAE: gallic acid equivalents. QE: quercetin equivalents. CE: catechin equivalents. UAE: Ultrasonic Assisted Extraction.

**Table 2:** Total phenolic, total flavonoid, and specified phenolic compounds concentrations in different apricot varieties and parts of fruits.

Cultivar	Variety	Fruit Part	Catechol (μg/g DM)	Catechin (μg/g DM)	Chlorogenic Acid (μg/g DM)	Caffeic Acid (μg/g DM)	p-Coumaric Acid (μg/g DM)	Ferulic Acid (μg/g DM)	Gallic Acid (μg/g DM)	TPC (mg GAE/100 g)	TFC (mg RE/100 g)	Reference
*Prunus armeniaca* L.	Çataloğlu	Pulp	13.62 ± 0.02	37.23 ± 0.02	111.71 ± 0.00	5.47 ± 0.00	14.55 ± 0.00	109.73 ± 0.04	15.76 ± 0.05	-	-	[66]
*Prunus armeniaca* L.	Çöloğlu	Pulp	6.72 ± 0.01	37.50 ± 0.01	221.81 ± 0.01	41.95 ± 0.00	127.92 ± 0.00	274.91 ± 0.01	31.62 ± 0.01	-	-	[66]
*Prunus armeniaca* L.	Hasanbey	Pulp	7.56 ± 0.03	32.16 ± 0.01	228.71 ± 0.01	80.08 ± 0.00	198.44 ± 0.01	295.09 ± 0.09	127.76 ± 0.01	-	-	[66]
*Prunus armeniaca* L.	Hacıhaliloğlu	Pulp	44.03 ± 0.02	111.62 ± 0.01	61.14 ± 0.01	21.67 ± 0.00	51.21 ± 0.02	220.39 ± 0.39	18.24 ± 0.08	-	-	[66]
*Prunus armeniaca* L.	Ordubat	Pulp	14.91 ± 0.01	8.84 ± 0.01	42.41 ± 0.01	4.96 ± 0.00	27.31 ± 0.01	93.24 ± 0.06	9.68 ± 0.04	-	-	[66]
*Prunus armeniaca* L.	Wilson delicious	Pulp	28.17 ± 0.01	199.55 ± 0.01	147.31 ± 0.00	117.10 ± 0.00	103.31 ± 0.01	110.66 ± 0.08	30.78 ± 0.07	-	-	[66]
*Prunus armeniaca* L.	Stark early orange	Pulp	7.76 ± 0.00	220.12 ± 0.01	93.86 ± 0.01	62.81 ± 0.00	105.54 ± 0.01	60.54 ± 0.08	14.96 ± 0.01	-	-	[66]
*Prunus armeniaca* L.	Harcot	Pulp	119.89 ± 0.01	177.32 ± 0.01	281.44 ± 0.01	167.86 ± 0.00	178.51 ± 0.00	251.33 ± 0.01	47.52 ± 0.02	-	-	[66]
*Prunus armeniaca* L.	Xiaobai Green mature	Peel	-	-	-	-	-	-	-	59.3 ± 1.5	76.7 ± 3.5	[68]
*Prunus armeniaca* L.	Xiaobai Green mature	Pulp	-	-	-	-	-	-	-	76.7 ± 3.5	27.9 ± 0.9	[68]
*Prunus armeniaca* L.	Xiaobai Full mature	Peel	-	-	-	-	-	-	-	48.5 ± 0.9	76.6 ± 1.8	[68]
*Prunus armeniaca* L.	Xiaobai Full mature	Pulp	-	-	-	-	-	-	-	28.5 ± 0.4	23.0 ± 1.3	[68]
*Prunus armeniaca* L.	Liguang Green mature	Peel	-	-	-	-	-	-	-	58.3 ± 2.5	81.9 ± 2.4	[68]
*Prunus armeniaca* L.	Liguang Green mature	Pulp	-	-	-	-	-	-	-	26.9 ± 1.4	23.2 ± 1.0	[68]
*Prunus armeniaca* L.	Liguang Full mature	Peel	-	-	-	-	-	-	-	56.9 ± 1.4	92.1 ± 2.5	[68]
*Prunus armeniaca* L.	Liguang Full mature	Pulp	-	-	-	-	-	-	-	28.4 ± 2.2	23.2 ± 0.6	[68]
*Prunus armeniaca* L.	Katy Green mature	Peel	-	-	-	-	-	-	-	50.1 ± 1.4	150.1 ± 7.5	[68]
*Prunus armeniaca* L.	Katy Green mature	Pulp	-	-	-	-	-	-	-	29.8 ± 1.2	31.3 ± 1.3	[68]
*Prunus armeniaca* L.	Katy Full mature	Peel	-	-	-	-	-	-	-	48.5 ± 0.7	143.1 ± 5.4	[68]
*Prunus armeniaca* L.	Katy Full mature	Pulp	-	-	-	-	-	-	-	24.2 ± 1.0	24.5 ± 0.8	[68]
*Prunus armeniaca* L.	Chuanzhihong Green mature	Peel	-	-	-	-	-	-	-	51.0 ± 2.4	137.5 ± 3.4	[68]
*Prunus armeniaca* L.	Chuanzhihong Green mature	Pulp	-	-	-	-	-	-	-	21.8 ± 0.9	19.9 ± 0.7	[68]
*Prunus armeniaca* L.	Chuanzhihong Full mature	Peel	-	-	-	-	-	-	-	45.1 ± 1.1	83.9 ± 1.7	[68]
*Prunus armeniaca* L.	Chuanzhihong Full mature	Pulp	-	-	-	-	-	-	-	16.6 ± 0.7	12.3 ± 0.2	[68]
*Prunus armeniaca* L.	Dajie Green mature	Peel	-	-	-	-	-	-	-	53.1 ± 1.3	110.4 ± 2.2	[68]
*Prunus armeniaca* L.	Dajie Green mature	Pulp	-	-	-	-	-	-	-	38.3 ± 0.9	63.4 ± 2.9	[68]
*Prunus armeniaca* L.	Dajie Full mature	Peel	-	-	-	-	-	-	-	43.8 ± 1.1	96.8 ± 3.0	[68]
*Prunus armeniaca* L.	Dajie Full mature	Pulp	-	-	-	-	-	-	-	24.7 ± 0.5	32.7 ± 1.1	[68]
*Prunus armeniaca* L.	Shushanggan Green mature	Peel	-	-	-	-	-	-	-	57.4 ± 3.6	73.5 ± 2.4	[68]
*Prunus armeniaca* L.	Shushanggan Green mature	Pulp	-	-	-	-	-	-	-	52.2 ± 2.5	54.9 ± 1.3	[68]
*Prunus armeniaca* L.	Shushanggan Full mature	Peel	-	-	-	-	-	-	-	51.5 ± 1.9	59.2 ± 0.7	[68]
*Prunus armeniaca* L.	Shushanggan Full mature	Pulp	-	-	-	-	-	-	-	39.7 ± 0.7	35.7 ± 1.4	[68]

DM: Dry matter, TPC: Total phenolic content, TFC: Total flavonoid content, GAE: Gallic acid equivalents, RE: Rutin equivalents.

**Table 3:** Total phenolic, flavonoid and anthocyanin contents of plum varieties in different fruit parts, along with the extraction methods.

Cultivar	Variety	Fruit Part	Extraction	Solvent	TPC	TFC	Total Anthocyanins (mg CGE/L)	References
*Prunus domestica* L.	Beltsville Elite B70197	Fresh plum	Ultra-sound	80% aqueous methanol	332 ± 3.1 mg GAE/100 g	237 ± 6.3 mg CE/100 g	-	[16]
*Prunus domestica* L.	Cacak Best	Fresh plum	Ultra-sound	80% aqueous methanol	319 ± 1.4 mg GAE/100 g	200 ± 2.5 mg CE/100 g	-	[16]
*Prunus domestica* L.	French Damson	Fresh plum	Ultra-sound	80% aqueous methanol	375 ± 3.8 mg GAE/100 g	215 ± 9.7 mg CE/100 g	-	[16]
*Prunus domestica* L.	Long John	Fresh plum	Ultra-sound	80% aqueous methanol	199 ± 2.5 mg GAE/100 g	126.3 ± 3.4 mg CE/100 g	-	[16]
*Prunus domestica* L.	Stanley	Fresh plum	Ultra-sound	80% aqueous methanol	174 ± 1.5 mg GAE/100 g	118 ± 2.6 mg CE/100 g	-	[16]
*Prunus domestica* L.	Yugoslavian Elite T101	Fresh plum	Ultra-sound	80% aqueous methanol	217 ± 4.9 mg GAE/100 g	146 ± 6.0 mg CE/100 g	-	[16]
*Prunus salicina* L.	-	Juice	-	-	3.77 ± 0.57 mg GAE/L	1990.8 ± 9.7 mg RE/L	1.0 ± 0.2	[84]
*Prunus salicina* L.	-	Juice	Ultra-sound	-	4.774 ± 0.27 mg GAE/L	2028.9 ± 13.2 mg RE/L	1.3 ± 0.1	[84]
*Prunus salicina* L.	-	Juice	Enzyme	-	6.381 ± 0.33 mg GAE/L	2153.9 ± 13.2 mg RE/L	9.1 ± 0.2	[84]
*Prunus salicina* L.	-	Juice	Ultra-sound and enzyme 15’	-	6.935 ± 0.47 mg GAE/L	2461.0 ± 3.7 mg RE/L	6.6 ± 2.3	[84]
*Prunus salicina* L.	-	Juice	Ultra-sound and enzyme 30’	-	6.517 ± 0.40 mg GAE/L	2240.7 ± 19.1 mg RE/L	7.6 ± 0.5	[84]
*Prunus salicina* L.	-	Juice	Ultra-sound and enzyme 60’	-	6.546 ± 0.55 mg GAE/L	2245.0 ± 3.7 mg RE/L	8.9 ± 0.5	[84]

TPC: Total phenolic content, TFC: Total flavonoid content, GAE: Gallic acid equivalents, RE: Rutin equivalents, CE: Catechin equivalents, CGE: Cyanidin-3-glycoside Equivalents.

**Table 4:** Total phenolic, flavonoid, anthocyanins and condensed tannins contents of sweet cherry varieties in different fruit parts along with the extraction methods.

Cultivar	Variety	Fruit Part	Extraction Solvent	Total Anthocyanins (mg CGE/100 g FW)	TFC	TPC (mg GAE/g)	Total Condensed Tannins (mg CE/g)	References
*Prunus avium* L.	Hongyan	Whole fruit pulp	Methanol	19.93 ± 0.14	7.97 ± 4.29 mg RE/100 g FW	-	-	[91]
*Prunus avium* L.	Caihong	Whole fruit pulp	Methanol	20.07 ± 0.11	43.55 ± 4.29 mg RE/100 g FW	-	-	[91]
*Prunus avium* L.	Rainier	Whole fruit pulp	Methanol	20.90 ± 0.27	77.27 ± 7.07 mg RE/100 g FW	-	-	[91]
*Prunus avium* L.	Burlat	Whole fruit pulp	Methanol	164.76 ± 5.41	253.32 ± 19.67 mg RE/100 g FW	-	-	[91]
*Prunus avium* L.	Lapins	Whole fruit pulp	Methanol	104.28 ± 1.84	140.01 ± 5.85 mg RE/100 g FW	-	-	[91]
*Prunus avium* L.	5–106	Whole fruit pulp	Methanol	147.38 ± 6.06	101.61 ± 12.25 mg RE/100 g FW	-	-	[91]
*Prunus avium* L.	Tieton	Whole fruit pulp	Methanol	126.06 ± 1.43	70.71 ± 9.73 mg RE/100 g FW	-	-	[91]
*Prunus avium* L.	Hongdeng	Whole fruit pulp	Methanol	142.74 ± 1.17	101.61 ± 2.81 mg RE/100 g FW	-	-	[91]
*Prunus avium* L.	Zaodaguo	Whole fruit pulp	Methanol	119.41 ± 0.99	120.34 ± 1.62 mg RE/100 g FW	-	-	[91]
*Prunus avium* L.	Van	Whole fruit pulp	Methanol	106.57 ± 1.50	68.84 ± 4.29 mg RE/100 g FW	-	-	[91]
*Prunus avium* L.	Bing	Whole fruit pulp	Ethanol	-	31 ± 5 mg QE/100 g	1.13 ± 0.04	0.03 ± 0.01	[92]
*Prunus avium* L.	Ron’s	Whole fruit pulp	Ethanol	-	51 ± 2 mg QE/100 g	0.87 ± 0.09	n.d	[92]
*Prunus avium* L.	Merchant	Whole fruit pulp	Ethanol	-	34 ± 2 mg QE/100 g	1.23 ± 0.01	0.04 ± 0.03	[92]
*Prunus avium* L.	Lapins	Whole fruit pulp	Ethanol	-	47 ± 1 mg QE/100 g	1.73 ± 0.90	0.17 ± 0.09	[92]
*Prunus avium* L.	-	Juice	-	-	58 g CE/100 g	0.60 ± 0.04	-	[93]

GAE: Gallic acid equivalents, RE: Rutin equivalents, CE: Catechin equivalents, CGE: Cyanidin 3-glucoside equivalents, QE: Quercetin equivalents, FW: Fresh Weight.

**Table 5:** Solvent and extraction methods were used to determine antioxidant activity, total phenolic content, total flavonoid content, and caffeic acid content in selected spices.

Spice	Solvent	Extraction Method	Antioxidant Activity (DPPH)	EC50	TPC	TFC	Caffeic Acid	References
*Origanum vulgare*	Aqueous Methanol 50%	Extraction	58.28 ± 0.33%	-	15.83 mg GAE/g	-	-	[107]
*Origanum vulgare*	Aqueous Methanol 80%	Shaker extraction 3 h	53.90 (TE)/g DW	-	44.08 mg GAE/g	5.13 mg QE/g		[108]
*Origanum vulgare*	Enzymes	Enzymatic	79.6 ± 2.04 μM trolox/100 g dw	-	-	-	649 ± 0.07 mg/100 g DM	[109]
Cinnulin PF	Aqueous	Extraction	-	0.015 mg/mL	82.3 mg GAE/g	-	-	[110]
*Cinnamomum cassia*	Aqueous Methanol 80%	Extraction 24 h	-	-	63.4 ± 0.021 mg GAE/g	-	-	[111]
*Cinnamomum zeylanicum *	Aqueous Methanol 80%	Extraction 24 h	-	-	119 ± 0.004 mg GAE/g	-	15.3 mg/100 g of DM	[111]
*Cinnamomum zeylanicum*	Methanol	Soxhlet 4 h	76%	-	-	-	-	[112]
*Cinnamomum zeylanicum*	Acetone	Soxhlet 4 h	66%	-	-	-	-	[112]
*Cinnamomum zeylanicum*	Ethyl acetate	Soxhlet 4 h	56%	-	-	-	-	[112]
*Cinnamomum zeylanicum*	Water	120 °C/20 min	94%	-	-	-	-	[112]
*Cinnamomum burmanni*/*Syzygium aromaticum* (1:1)	Ethanolic extract of grape origin (EEGO)	Extraction 24 h	55.12 ± 0.01	0.08 ± 0.01 mg/L	1120.24 ± 0.01 mg GAE/L		634.65 ± 0.01 mg/L	[98]
*Syzygium aromaticum*	Ethyl acetate	Extraction 60 min	25.3%	-	58.8 mg GAE/g extract	4.70 mg QE/g extract	-	[113]
*Syzygium aromaticum*	Ethanol 80%	Extraction 60 min	Approx. 90%	-	293 mg GAE/g extract	12 mg QE /g extract	-	[113]
*Syzygium aromaticum*	Water	Extraction 60 min	91.4%	-	230 mg GAE/g extract	17.5 mg QE/g extract	-	[113]
*Syzygium aromaticum*	Ethanol	Extraction	93%	-	-	-	-	[114]
*Syzygium aromaticum*	Ethanol 70%	Extraction 24 h	95%	0.004 mg/mL	188.35 mg EE/g	-	-	[115]
*Syzygium aromaticum*	Ethanol 80%	Extraction 24 h	95%	0.037 mg/mL	250.93 ± 1.33 mg GAE/ g extract	-	-	[116]
*Crocus sativus* L. Greece	Methanol	Extraction 24 h	27.41 ± 1.57%	-	68.26 ± 31.82 mg GAE/g DM	-	-	[117]
*Crocus sativus* L. Iran	Methanol	Extraction 24 h	39.49 ± 1.05%	-	101.93 ± 11.11 mg GAE/g DM	-	-	[117]
*Crocus sativus* L. Morocco	Methanol	Extraction 24 h	24.56 ± 0.87%	-	62.93 ± 21.33 mg GAE/g DM	-	-	[117]
*Crocus sativus* L. Spain	Methanol	Extraction 24 h	26.15 ± 1.70%	-	43.70 ± 11.58 mg GAE/g DM	-	-	[117]
*Crocus sativus* L.	Water	Extraction 1 h	-	1.227 ± 0.05286 mg/mL	104.82 ± 4.36 mg GAE/g DM	26.88 ± 2.60 mg QE/g DM	-	[118]
*Crocus sativus* L.	Methanol	Extraction 48 h	-	1.3648 ± 0.09562 mg/mL	114.66 ± 3.48 mg GAE/g DM	31.27 ± 0.73 mg QE/g DM	-	[118]
*Crocus sativus* L.	Aqueous ethanol	Soxhlet 6 h	-	0.88877 ± 0.08736 mg/mL	130.39 ± 13.79 mg GAE/g DM	39.06 ± 10.85 mg QE/g DM	-	[118]
*Crocus sativus* L.	Diethyl ether	-	-	0.30944 ± 0.0333 mg/mL	214.29 ± 12.68 mg GAE/g DM	84.46 ± 16.10 mg QE/g DM	-	[118]
*Crocus sativus* L.	Ethyl acetate	-	-	0.7211 ± 0.03 mg/mL	154.32 ± 14.03 mg GAE/g DM	46.88 ± 4.80 mg QE/g DM	-	[118]
*Crocus sativus* L.	n-Butanol	-	-	0.884 ± 0.1526 mg/mL	171.8 ± 13.41 mg GAE/g DM	60.59 ± 11.38 mg QE/g DM	-	[118]

TE: Trolox Equivalents, GAE: Gallic Acid Equivalents, QE: Quercetin Equivalents, EE: Eugenol Equivalents, DM: Dry Matter.

## Data Availability

The manuscript contains all the relevant data.
